# Weighted false discovery rate controlling procedures for clinical trials

**DOI:** 10.1093/biostatistics/kxw030

**Published:** 2016-07-21

**Authors:** Yoav Benjamini, Rami Cohen

**Affiliations:** Department of Statistics and Operations Research, The Sackler Faculty of Exact Sciences and The Sagol School for Neurosciences, Tel Aviv University, Tel Aviv 39040, Israelybenja@tau.ac.il; Department of Statistics and Operations Research, The Sackler Faculty of Exact Sciences, Tel Aviv University, Tel Aviv 39040, Israel

**Keywords:** FDR, Gatekeeper procedures, Hierarchical testing, Primary endpoints, Secondary endpoints

## Abstract

Having identified that the lack of replicability of results in earlier phases of clinical medical research stems largely from unattended selective inference, we offer a new hierarchical weighted false discovery rate controlling testing procedure alongside the single-level weighted procedure. These address the special structure of clinical research, where the comparisons of treatments involve both primary and secondary endpoints, by assigning weights that reflect the relative importance of the endpoints in the error being controlled. In the hierarchical method, the primary endpoints and a properly weighted intersection hypothesis that represents all secondary endpoints are tested. Should the intersection hypothesis be among the rejected, individual secondary endpoints are tested. We identify configurations where each of the two procedures has the advantage. Both offer higher power than competing hierarchical (gatekeeper) familywise error-rate controlling procedures being used for drug approval. By their design, the advantage of the proposed methods is the increased power to discover effects on secondary endpoints, without giving up the rigor of addressing their multiplicity.

## 1. Introduction

The practice of clinical research trials spans a wide spectrum. On one extreme, we have Phase III clinical trials for drug registering that are regulated by the relevant national or international agencies. These enjoy clearly specified population of study, treatments to be tested, and prespecified statistical methods for the analysis and reporting of results. In particular the endpoints by which success is determined are specified and if more than one is specified, controlling for the possible effect of selection is appropriately addressed by methods that offer the control of making even one false discovery (familywise error-rate). Details of the code used for the statistical analysis are kept, allowing the skeptic inspector to verify that the analysis is reproducible, from raw data to report’s figures. Moreover, a recent European initiative intends to further increase the transparency of the process by allowing open access to the data of such trials. At the other extreme we have the loosely defined protocols, multiple ways of defining success, questionable statistical practices, and the concern that only findings that are significant at the customary 0.05 level are reported or emphasized by selection into the abstract. Such practices, and particular the last one have caused the concern exemplified in Ioannidis (2005) “Why most research findings are false.” It has been the focus of a discussed paper in this journal on the estimation of the sciencewise false discovery rate (FDR) in medical research (Jager and Leek [2014] and its discussions).

In their discussion, Benjamini and Hechtlinger (2014) noted that when we move away from the regulatory studies, even in studies appearing in top publishing venues, selective inference is compromised. We studied in depth a sample of 100 papers that were published between the years 2000 and 2010 in the *New England Journal of Medicine*. (See references to these papers at Section 1 of supplementary material available at *Biostatistics* online). All papers addressed multiple endpoints, but in 80% of them the issue of multiplicity was entirely ignored. In the remaining 20%, the issue of multiplicity was but partially addressed. Moreover in another part of the discussion of Jager and Leek (2014), it was shown that significant findings, even though unadjusted, more often find their way to the abstract than non-significant ones. As is the case in many fields of research, it is the abstract that is intended to disseminate the important information to the community of medical practitioners. On another note, we have that 84% of the aforementioned papers specify *only one single *primary endpoint, so the main focus of the current paper is the case of one primary endpoint and multiple secondary ones.

As a concrete example, Natalizumab was examined by Ghosh *and others* (2003) for the treatment of Crohn’s disease. Four measures of success were compared at five different time points between a placebo-controlled group and three different treatment groups, each prescribed to a different dose, and a fourth one was measured at two time points. The study defined one primary endpoint clinical remission on Crohn’s Disease Activity Index at week 6 with two infusions of 6 mg per kilogram, and 50 secondary endpoints among the remaining. No reference was made in the trial to the multiplicity of tested hypotheses; both the single primary endpoint and the secondary endpoints were considered significant at *p* $<$ 0.05 (and referred to as such in their discussion). Consequently, even though the primary endpoint was not rejected the study considered 27 hypotheses as yielding discoveries. In contrast, applying gatekeeper approaches (e.g. Dmitrienko *and others*, 2003, 2008; Guilbaud, 2007) that are used in regulatory research to control for the familywise error-rate when inference is sought for both the primary and secondary endpoints would have yielded no discoveries at all. Indeed, the main reason for dropping multiplicity adjustment is the loss of power that comes with it, and the increased number of patients therefore needed.

The above example is that of a Phase II trial. At this and earlier phases of clinical research, the recommended treatment and the way to measure its success have not stabilized yet, and a number of options are still explored. The goal is therefore not to simultaneously infer on all comparisons made, but rather to selectively infer on the most promising ones. The use of FDR approach allows us to explore alternatives to the currently defined primary endpoint. The use of weights in the FDR criterion (Benjamini and Hochberg, 1997) can reflect the varying importance of the tested endpoints, which underlies their partition to primary and secondary; the rejection of a highly weighted hypothesis carries more importance than a low weighted one, but at the same time if it is rejected in error—the error counts more.

In view of the above considerations, in this paper we propose the use of the weighted procedures, and in particular a new hierarchical procedure that controls the weighted FDR (Benjamini and Hochberg, 1995) while considering their division to primary and secondary (as the gatekeeper methods do). We try to strike a balance between these two extremes. In the above example the proposed hierarchical weighted method revealed that 12 endpoints should be treated as discoveries (see Section 3.3), and the single-level weighted one revealed only 10 discoveries. The failure of the primary endpoint result raised the importance of secondary endpoints, and emphasized their proper analysis in face of selection, leading to the subsequent possibility of transforming one of them into a primary endpoint, which will remain significance in Phase III.

## 2. The weighted FDR controlling procedures

### 2.1. The criterion

Assign to each of the hypotheses $H_i$ being tested a weight $w_{i} \ge 0, i=1,2,\dots m$. Without loss of generality, let $\sum w_i=m$. Define $R_i=1$ if $H_{i}$ is rejected, and otherwise $R_{i}=0$; Define $V_i=1$ if $H_{i}$ is erroneously rejected, and otherwise $V_i=0$. Let

(2.1) $$Q(w)=\{\begin{array}{ll} \frac{\sum_{i=1}^{m}w_{i}.V_{i}}{\sum_{i=1}^{m}w_{i}.R_{i}}, & \sum_{i=1}^{m}\;w_{i}\;R_{i}>0,\\ 0, & \text{Otherwise,} \end{array}$$

then $\mathrm{wFDR}=E(Q(w))$ is the *weighted FDR* which we wish to control at level $q$.

Given $P$ primary endpoints to be tested with corresponding p-values $\mathbf{p}_p=(p_{\mathrm{p}_1}, \cdots, p_{\mathrm{p}_i},\cdots, p_{\mathrm{p}_p}),$ and $S$ secondary endpoints with corresponding p-values $\mathbf{p}_s=(p_{\mathrm{s}_1}, \cdots, p_{\mathrm{s}_i},\cdots, p_{\mathrm{s}_p})$, let $\mathbf{W_p}$ and $\mathbf{w_s}$ be the corresponding vectors of weights, with $w_{pi}$ and $w_{sj}$ denoting the weights assigned to the *i*th primary and the *j*th secondary endpoints, respectively. For ease of notation, we require

(2.2) $$\sum w_{s_{i}}+\sum w_{p_{i}}=S+P.$$

In the procedures below normalization of weights to satisfy the above need not take place. We shall study two regimes of weights. In the first min$(w_{P_i})\ge$ max$(w_{\mathrm{s}_j})$, reflecting the fact that any primary endpoint is more important than any secondary one. In the second regime,

(2.3) $$R^{\prime }=\frac{\sum W_{P_{i}}}{\sum W_{S_{j}}}\geq 1.$$

Under (2.3) not only does each primary endpoint get a weight larger than of a secondary endpoint, but so does the combined weight of the primary endpoints relative to the combined weight of the secondary ones. We set as our goal to control the weighted FDR of the combined set of primary and secondary endpoints hypotheses at level $q$.

### 2.2. The weighted FDR controlling procedure

(Benjamini and Hochberg, 1997)

(i) Sort all p-values, $p_{(1)}\le \cdots p_{(i)}\le\cdots\le p_{(m)}$ where each $p_{(i)}$ corresponds to hypothesis $H_{(i)}$ with its assigned weight $w_{(i)}$.
(ii) Let $k={\rm max} \left\lbrace j: p_{(j)}  \le \sum\limits_{i=1}^j w_{(i)}.q/m \right\rbrace.$
(iii) Reject hypothesis $H_{(1)} \cdots H_{(k)}$ if such $k$ exists; otherwise reject none.

We shall refer to the above procedure as wBH, or $wBH_q(\mathbf{w,p})$ when applied at level $q$ to the vector of p-values $\mathbf{p}$, with the vector of weights $\mathbf{w}$. It controls the weighted FDR at level $q$ multiplied by the ratio of the sum of weights corresponding to true null hypotheses to m, which is always smaller than $q$, for independent and positive regression dependent test statistics (Benjamini and Hochberg, 1997; Benjamini and Kling, 1999).

### 2.3. The hierarchical weighted FDR controlling procedure

The hierarchical structure is used in multiple testing to reflect logical implications between hypotheses (e.g. Shaffer, 1995), or to reflect an existing or sometimes desirable inference process. The flow of inference can be structured in a tree-like process, with each rejected hypothesis may be followed with further inferences, but non-rejected hypotheses are not followed. (Examples of the latter approach can be seen in Yekutieli *and others* [2006] and Benjamini and Heller [2008].) Hierarchical testing of the latter form has increased power due to the grouping of similarly behaving parameters, by offering two advantages: The increased sensitivity of the group level test statistic and the reduction in the number of hypotheses tested in the first stage.

#### 2.3.1. The hierarchical weighted FDR controlling procedure

(i) Sort the p-values of secondary hypotheses $p_{\mathrm{s}_{(1)}} \le \cdots p_{\mathrm{s}_{(i)}} \cdots p_{\mathrm{s}_{(s)}}.$
(ii) Calculate p-value of their intersection hypothesis $H^*=\cap H_{S_i} $ using the weighted Simes test statistics (Benjamini and Hochberg, 1997; Simes, 1986):
  $$p^{*}=min_{i}\;\;p_{s_{\left(i\right)}}.\frac{\sum_{j=1}^{S}w_{s_{j}}}{\sum_{j=1}^{i}w_{s_{\left(i\right)}}},$$
  and assign it the sum of the weights of the secondary hypotheses, $w^*=\sum w_s{_i}$.
(iii) Pool $p^*$ and its weight $w^*$ together with the p-values of the primary hypotheses and their weights, to get $\mathbf{p'}=(p^*,\mathbf{P_{p}})$ and $\mathbf{w'}=(w^*,\mathbf{w_{p}})$. Apply the $wBH_{\alpha}(\mathbf{p',w'})$ procedure and reject any primary endpoint among the rejected.
(iv) If the intersection hypothesis $H^{*}$ is among the rejected, test the secondary endpoints by the $wBH_{\alpha}(\mathbf{p_s}, w_s)$ procedure (2.2); if the intersection hypothesis is not rejected - no secondary endpoint hypothesis.

The procedure is presented in its generality, even though our interest in this paper is only with one primary endpoint and all secondary ones receiving equal weights, namely $P=1$ and $w_{\mathrm{s}_i}\equiv w_s$. $R\ge 1$ denote the weights ratio, which is the ratio between the weight of the primary hypothesis and the weight of each secondary hypothesis. We emphasize the case of equal weight for all secondary endpoints as this reflects the current categorizing of endpoints to primary and secondary endpoints. At the end of this paper, we refer briefly to the case of non-equal weights for the secondary endpoints.

The above procedure should be applied at a level lower than $q$ to assure $\mathrm{wFDR} \le q$. The required $\alpha$-level is a function of the parameters $\alpha(q,P,S,R')$ as well as the assumed dependency structure among the p-values. In Section 4 we analyze *a(q,P,S,R’)* for independent and positively dependent test statistics, and offer expressions for upper bounds. These are numerically implemented as a part of the HWF algorithm in Section 2 of the supplementary material available at *Biostatistics* online and implemented in a webpage http://spark.rstudio.com/shayy/HWBH/.

It is important to note that although the rejection of the intersection hypothesis is considered for the wFDR at step 3, it does not enter directly into the weighted FDR we wish to control.

Also note that the primary endpoint is always tested at a level less than $\alpha$. For our case of interest $P=1$, there are only two hypotheses in the first level (step 3); the $wBH$ used at this level (which is equivalent to the weighted Simes test) controls the probability of making an erroneous rejection of the primary hypothesis at level $\le \alpha$.

## 3. The analysis of the Natalizumab and Posaconazole trials

We now demonstrate the use of the proposed procedures in the analysis of two clinical trials. In both $P=1$, and we use equal weight for the secondary endpoints, $W_{\mathrm{s}_i}\equiv W_s$.

Example 3.1 We first return to the Netalizumab experiment discussed in the introduction. There are $S=50$ secondary endpoints. We choose to use $R'=2$, namely $R=100$, For the HWF procedure, the first stage requires testing p-values of both the intersection hypothesis $p^*$ and the primary endpoint ($p_p$) against matching critical values. For calculating $p^*$ we begin with the list of sorted secondary endpoints p-values: $p_{S_{(1)}}=4.77\times 10^{-5}, p_{S_{(2)}}=6.29\times 10^{-5}, p_{S_{(3)}}=1.44\times 10^{-4}, \cdots,p_S{_{(50)}}=0.992$ (the full list is available at Section 3 of supplementary material available at *Biostatistics* online). $p^*=min  \left(p_{S_{(i)}}\cdot \frac{S}{i}\right)=0.00157$. For $q=0.05, P=1, S=50$, and for the chosen $R'=2, \alpha(0.05,1,5,2)=0.0257$. Now the primary endpoint p-values $P_P{_1}=0.533 > \alpha= 0.0257$ and so it is not rejected, while the intersection hypothesis p-values $p^*=0.00157 \le \alpha w ^* / (w^*+w_p)=\alpha/(1+R')=0.0086$ and hence rejected. Turning to the secondary endpoints, the largest secondary endpoint’s p-value below the critical value $\alpha \cdot i/S$ $p_{S_{(12)}}=0.006\le \alpha \cdot i/S=0.0257.12/50=0.00617$ is the 12th: Thus, according to the level, 0.05 HWF procedure the 12 secondary endpoints with p-values lower than 0.006 can be rejected.

Using the wBH procedure the largest $i$ for which $P_{S_{(i)}}\le 0.05 \cdot (i/50)/3$ is $i=10$, so 10 secondary endpoints are rejected.

Example 3.2 Posaconazole was tested against Fluconazole regarding the prevention of infection (Ullmann et al. 2007). The primary endpoint was incidence of invasive fungal infections till day 112. Six secondary endpoints were tested (listed here according to their sorted p-values): (i) breakthroughs of invasive fungal infection of particular type (aspergillosis) during the exposure period, (ii) breakthrough of invasive fungal infections, (iii) breakthrough of invasive fungal infections of particular type (aspergillosis) during exposure period, (iv) mortality from fungal infection, (v) time to occurrence of an invasive fungal infection, and (vi) overall mortality.

The p-values were: $P_p{_{1}}=0.07; P_{S_{(6)}}>0.5, P_{S_{(5)}}=0.048, P_{S_{(4)}}=0.046,P_{S_{(3)}}=0.006, P_{S_{(2)}}=0.004, {\rm and} P_{S_{(1)}}=0.001$. Note that the primary endpoint was first tested for non-inferiority, but as this was defined in the protocol as preliminary condition for further analysis the p-value above is valid for testing superiority.

Using HWF with $R=3$, we sort the secondary endpoints’ p-values and get the intersection hypothesis p-value $P^*= {\rm min}  \left(p_{S_{(i)}}\cdot \frac{6}{i}\right)=0.001*6/1=0.006$. Its weight is $w^*=6/9$ while $w_p=3/9$. Since $P^*\le P_{P_i}$, the secondary endpoint is tested if $P^*\le 0.0317*\frac{6}{6+3}=0.0212$, which is the case. So the largest $P_{S_{(i)}}\le 0.0317\cdot 1/6$ is for $P_{S_{(3)}}=0.006$, and all three secondary hypotheses regarding reduced incidence of special types of infection are rejected.

Using wBH, the largest $P_{S_{(i)}}> P_{P_1}>0.05$ so only smaller $p_{S_{(i)}}$ can be rejected. The $p_{S_{(i)}}$ are compared with $0.05 \cdot i/(6+3)$, and again $p_{S_{(3)}}$ is the largest one satisfying it, leading to the same conclusion.

Note that any use of gatekeeping method will lead to no rejection of any secondary. In contrast not adjusting for the multiplicity of endpoints, which was the way the analysis was done, has led to statements about the advantage of Posaconazole in terms of reduced mortality from fungus infections and increased time to infection, both barely reaching significance at the 0.05 level.

## 4. The maximal wFDR of the hwf procedure

### 4.1. Finding the worst case for independent test statistics with $R \ge S$

For ${R \ge S}$ and $S > 2$, we show that the worst case array (WCA), where ${\mathrm{wFDR}}$ is maximized, is achieved by assigning one of the secondary p-values, say the first, a value that assures the opening of the hierarchy, i.e. $p_{\mathrm{s}_i}\equiv p \le \alpha / (R+S)$, and assign all others $p_{\mathrm{s}_i}\sim U(0,1)$ and $p_{\mathrm{p}_1}\sim U(0,1)$.

The proof has two parts. We first show that for a single false null secondary hypothesis indeed the value is maximized when $p_s{_1}=p' \le \alpha /(R+S)$. We then show that when the number of false hypotheses is $>1$, the maximal *wFDR* is even lower than the lower bound in case WCA. Now, under WCA it can be exactly calculated:

(4.1) $$\begin{array}{ll} \text{HWFDR}={\left(1-\alpha \right)}^{2}\sum_{k=0}^{p}\sum_{j=0}^{s-1}\left(\begin{array}{c} p\\ k \end{array}\right) & {\left(\alpha \frac{s+RK}{s+Rp}\right)}^{k}{\left(1-\alpha \frac{s+Rk}{s+Rp}\right)}^{\left(p-k-1\right)}\\ & \;\times \left(\begin{array}{c} s-1\\ j \end{array}\right){\left(\alpha \frac{j+1}{s}\right)}^{j}{\left(1-\alpha \frac{j+1}{s}\right)}^{s-j-2}\left(\frac{Rk+j}{Rk+j+1}\right), \end{array}$$

wFDR turns out to be bigger than $q$, but $\alpha$ can be calculated so that $\mathrm{HWFDR} \le q$.

For the case $S=2$ the worst configuration is when all hypotheses are true, and $\mathrm{HWFDR}\le q$ with no further modification. For these proofs, see Section 4 of supplementary material available at *Biostatistics* online.

### 4.2. Finding the worst case in for independent test statistics with R$<$S

For ${1 \le R \le S}$, the above bound for one WCA still holds, but it cannot be shown that when the hypotheses for two or more secondary endpoint are false the upper bound is not always lower. It is so for $S<20$, but it misses for larger S by an amount that increases as R decreases. For $R=1$ and $S=50$, 100, 1000, 10,000 the differences are 0.00098, 0.00129, 0.00156, and 0.00159 and remains so as S further increases. While the direct proof used in 4.1 does not work, simulations at such scenarios show that actual wFDR is lower than the upper-bound in (4.1).

### 4.3. Finding the worst case in for dependent test statistics

The case of independence is important from a theoretical point of view as a close bound can be offered. In practice, the multiple endpoints are measured on the same subjects and therefore are likely to be positively dependent. In a case of positive regression dependency (Benjamini and Yekutieli, 2001) between the test statistics, and for $S > 2$,

(4.2) $$HWFDR\leq (1-\alpha )\cdot \frac{\alpha (S-1)}{S}+\alpha \left[\left(\alpha \frac{S-1}{S}\right)+\left(1-\alpha \frac{S-1}{S}\right)\frac{R}{R+1}\right].$$

For the special case when $S=2$ and $1 \le R \le 3.15$, and when $S=3$ and $1 \le R \le 1.28$, and furthermore all hypotheses are true, the above bound does not hold. A higher but simpler bound is

(4.3) $$HWFDR\leq \alpha [1+RS/(R+S{)}^{2}].$$

Note: we rely on this bound throughout the rest of this work even though there is a strong support that in this particular case, as well as for other values of R and S, when all hypotheses are true the bound is actually $\alpha$ (Benjamini and Heller, 2008). For proofs of the above bounds see Section 5 of supplementary material available at *Biostatistics* online).

## 5. Power comparisons

The power of the HWF procedure is compared to the power of the single-level weighted BH and the gatekeeper procedures. The power, relevant only if some tested hypotheses are false, may be defined in various ways. The comparison of power is made through simulation.

### 5.1. Compared power measurements

(i) Overall weighted power is computed on the basis of both primary and secondary endpoints, each assigned its weight. This power is thus defined as: $\Pi_G=E\left(\sum_{i\epsilon I_1} w_i \cdot R_i /\sum_{i\epsilon I_1} w_i \right)$, where $I_1$ the group of all false endpoints.
(ii) For the primary endpoint, let $R_0=1$ if it is false and rejected and otherwise $R_0=0$, then the power is $\Pi_P=E(R_0)$.
(iii) For the secondary endpoints alone, the power is: $\Pi_S=E\left(\sum_{i\epsilon I_1} w_i \cdot R_i/ \sum_{i\epsilon I_{\mathrm{s}_1}} w_i \right)$, where $I_{S1}$ is the group of all false secondary endpoints.
(iv) For discovering at least one secondary endpoint, let $R_{\rm ALOS}=\left\{\begin{array}{@{}ll@{}} 1 & \sum_{i\epsilon I_{S_1}} R_i >0\\ 0 & \text{Otherwise}\end{array}\right\}$ and define $\Pi_{\rm ALOS}=E(R_{\rm ALOS})$.
(v) We also considered a combination of $\Pi_P$ and $\Pi_S$, $\Pi_{\delta}=\frac{\delta}{\delta+1}\Pi_P+\frac{1}{\delta+1}\Pi_s$ in which $\delta$ reflects the relative importance of the two measures of success. It will serve us in the practical discussion of the recommended procedure.
  Gate Keeper (GK) procedures were simulated only for $S$ values of 2, 4, 8 as significance levels for the different endpoints require the computation of p-values for each possible intersection hypothesis, a number that increases exponentially with the number of endpoints. Power was computed on the basis of 10 000 simulations for each of the 12 880 different value configurations. The greatest standard deviation measured for the power average difference between the HWF procedure compared to the wBH procedure amounted to 0.0018.

### 5.2. Simulation parameters

1. The effect of the primary endpoint, $\mu_{\rm p}$, for the values: 0, 1, 2, 3, 4.
2. $R=1, 2, 8, 32, 128, 512$.
3. The number of secondary endpoints, $S=2, 4, 8, 16, 32$.
4. The proportion of false secondary endpoints, $m_{1S}/S=0.25, 0.5, 0.75, 1$ (for $S=2$ no calculation was performed for the values 0.25 and 0.75).
5. The effect of one of the false secondary endpoints denoted by $\mu_{\rm s}=2, 3, 4, 5, 6$ and of the remaining false secondary endpoints $\mu_{{\rm s}i}=1, 1.5, 2, 2.5, 3$.

The test statistic for the primary endpoint is $X_i=Z_0+\mu_p$ while test statistics for the secondary endpoints are $X_i=Z_i+\mu_i \,\forall \, i=1,\cdots,S$, where $Z_i \sim N(0,1)\,\forall\, i=0,\cdots,S$ and $Z_i$ are independent. For the $i$th endpoint, the two-tailed test is: $H_{0i}:\mu=0, H_{1i}:\mu\ne 0$.

### 5.3. Analyzing the simulated results

**Fig. 1.**
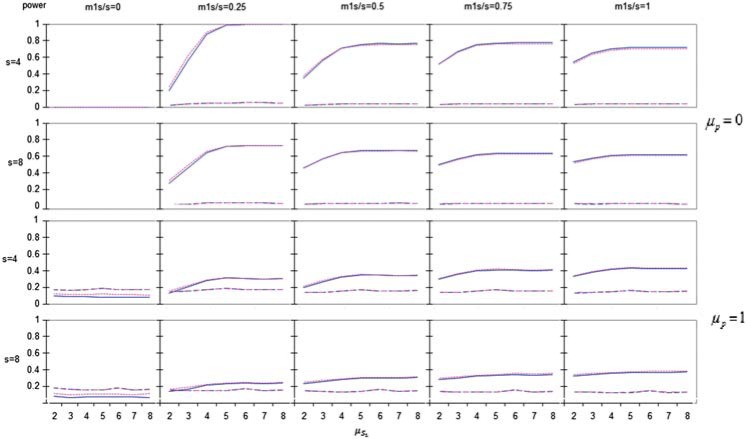
Overall power versus secondary endpoint’s parameter $\mu_{S_1}$. In the first two rows $R' = 1$, $\mu_p = 0$ (which is actually power for secondary), so the overall power of the GK procedure is bounded by $\alpha$ while the HWF and wBH procedures have much higher power for all different parameter values. This remains also true even when the primary endpoint has but a small effect (last two rows) where $\mu_p = 1$. Continuous blue line = HWF; dotted pink = wB-H; dashed blue = Simes GK; dashed pink = Bonferroni GK.

(i) With no effect at the primary endpoint (i.e. $\mu_p=0$), the overall power of the GK procedure is bounded by $\alpha$ while the HWF and wBH procedures have much higher power for all different parameter values (see upper two rows of Figure 1 for $R'=1$, i.e. ${R=S}$).
(ii) This remains also true even when the primary endpoint has but a small effect ($\mu_p=1$) as seen in the lower two rows of Figure 1, except when all secondary endpoints have no effect. The same holds for higher $\mu_p$ as well (see Figure S1 for $\mu_p=3$ in supplementary material available at *Biostatistics* online).

**Fig. 2.**
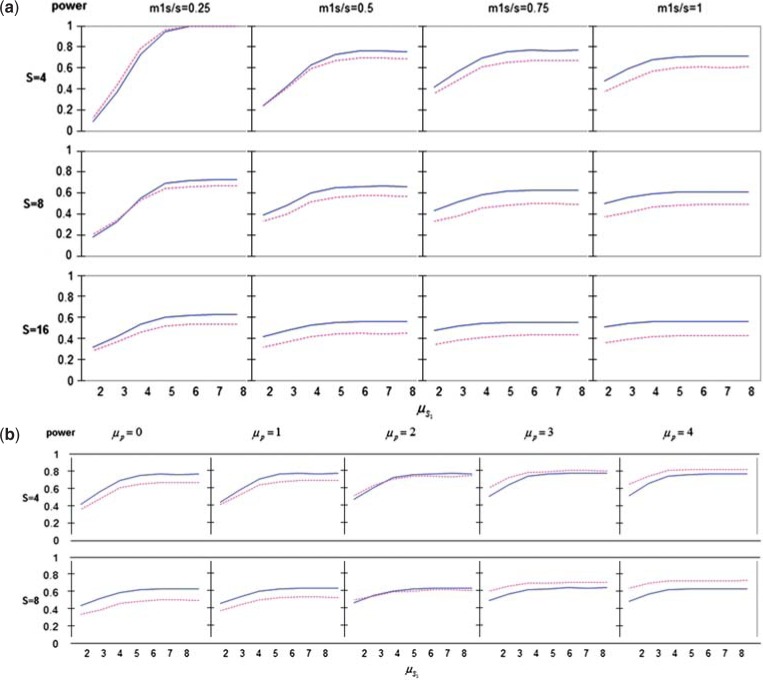
(a) Overall power versus secondary endpoint’s effect $\mu_{S_1}$ where $R' = 10$, $\mu_p = 0$. (b) Power for secondary endpoints versus secondary endpoint’s effect $\mu_{S_1}$. This shows similar trends to those in the upper two rows of (a), but in this setting the difference in power between the two weighted procedures becomes evident, and the HWF has higher power. Continuous blue line = HWF; dotted pink = wB-H; dashed blue = Simes GK; dashed pink = Bonferroni GK. $R' = 10$, $\frac{m_1S}{S} = 0.75$, $\mu_p$ = 0, 1, 2, 3, 4. When the primary effect is low the HWF procedure is more powerful than the wBH in discovering secondary endpoints. When it is high the opposite is true. Continuous blue line= HWF; dotted pink = wBH. For $R$ between 1 and 10 and d assigning more weight to the power for the primary endpoint, HWF is superior. This remains true for $R$ closer to 1 even when the weight given to the secondary is more than eight times more than to the primary. For $R$ bigger than 10 the wBH is more powerful. In this setting unless $R$ is larger than 16 ($R' > 1$) and d assigning more weight to the power for the secondary, an unlikely setting, the wBH is superior.

Figure 2(a) shows that when the ratio $R'$ is high, and $\mu_p=0$, the power of the HWF procedure tends to be greater than that of the wBH procedure, and the advantage increases as $m_{1S}/S$ increases and as $S$ increases. The power curves in Figure 2(a) stabilize once the effect of one of the secondary is above 4, as the hierarchy is bound to open.

Figure 2(b) presents the rejection power for the secondary endpoints as a function of one of the secondary’s effect and the effect of the primary endpoint, where $R'=10$. It is apparent that as $\mu_p$ decreases the difference in rejection power of secondary endpoints increases in favor of the HWF procedure as compared to the wBH procedure. When $\mu_p$ values are small, and the weight of the primary endpoint is high, the critical values for the secondary endpoints’ p-values are lower, lowering their probability of being rejected. In contrast, with the HWF procedure, once a hierarchy opens, the value of $R'$ is no longer involved in determining the critical values for the secondary endpoints, hence stems its advantage in power. For $\mu_p=2$, the power difference diminishes between the two procedures for the various parameter values (remaining low for other values of $R'$ as well). With higher effect of the primary endpoint, $\mu_p=3, \mu_p=4$, the power achieved under the wBH procedure is greater than that achieved under the HWF procedure. For these larger $\mu_p$, the p-values of the primary endpoint is small, the wBH procedure will set higher critical values for the secondary endpoints, so the power of secondary endpoints is larger than that of the HWF.

When $\mu_p$ is large, the rejection power of secondary endpoints decreases in importance. It is more important to achieve high rejection power of secondary endpoints when the rejection probability of the primary endpoint is low, as is the case where $\mu_p$ is small, and in this regime the HWF procedure’s advantage is clear.

## 6. Practical recommendation

**Fig. 3.**
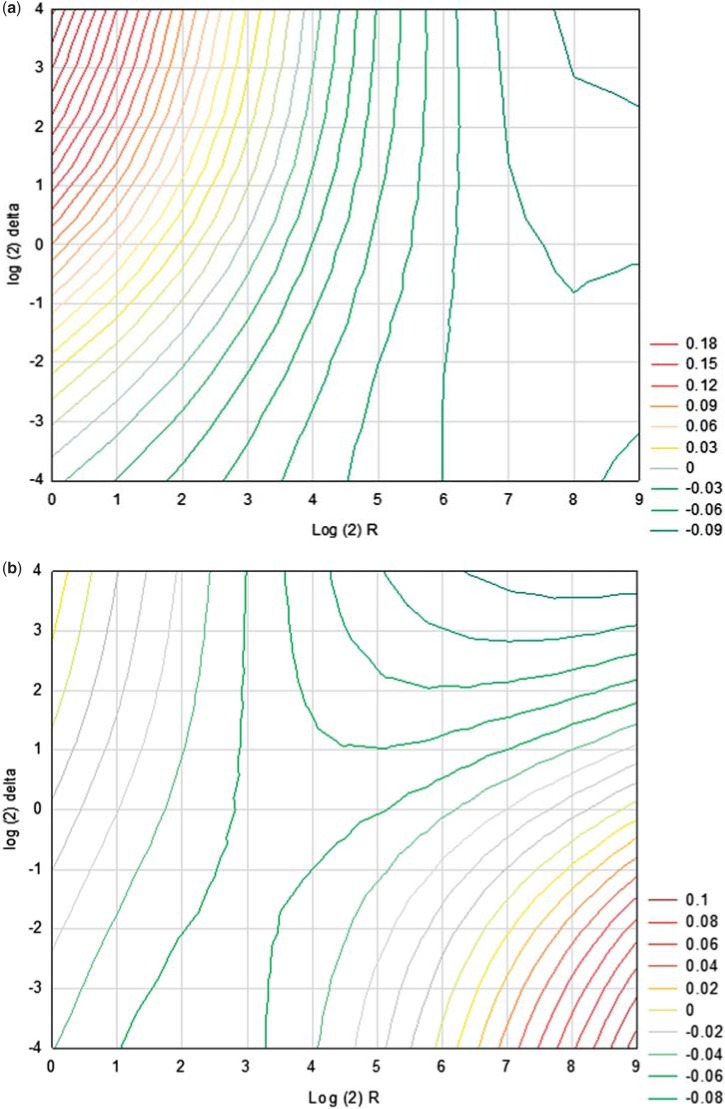
The difference in power as a function of $R$ and $\delta$: (a) HWF weighted power = wBH weighted power for $\mu_p =2$, $S=16$, $m_1S/S=0.25$, $\mu_S=1.5$, $\mu_{S_1}=6$ and (b) HWF weighted power – wBH weighted power for $\mu_p=2$, $S=16$, $m_1S/S=0.75$, $\mu_S=3$, $\mu_{S_1}=6$.

Note that the practice used until the concern about the multiplicity of the secondary endpoints was raised can be well described as a wBH procedure with $1/R =0$: If the primary is rejected at 0.05 level, any secondary that will be below 0.05 is also rejected, without any regard to their multiplicity; If the primary is not rejected none of the secondary can be rejected as well. Interestingly, the HWF in this case will result with a different procedure in the style of gate-keeper procedures: $\alpha' \sim \alpha$, so if the primary is rejected at 0.05 level the secondary endpoints are tested using the BH at level 0.05; If the primary is not rejected none of the secondary can be rejected as well. Of course less extreme choices need be considered. In Figures 3(a) and (b) we further introduce $\delta$, the relative weight of the primary and the secondary endpoints in the combined power, to shed light on the decision in practical situation. Note that $\delta$ should be considered the design stage and does not enter the analysis.

The difference in power as a function of $R$ and $\delta$, HWF weighted power $-$ wBH weighted power, is displayed in Figures 3(a) and (b). In both $\mu_p=2$, and $S=16$, $\mu_{S_1}=6$. In Figure 3(a), $m_1S/S=0.25$, $\mu_S=1.5$, representing fewer and weaker secondary effects (except for one). For $R$ between 1 and 10 and $\delta$ assigning more weight to the power for the power of the primary to HWF is superior. This remains true for $R$ closer to 1 even when the relative weight given to the secondary over the primary is more than 8. For $R$ bigger than 10 the wBH is more powerful.

In Figure 3(b), $m_1S/S=0.75$, $\mu_S=2$, representing more and stronger secondary effects. In this setting unless $R$ is larger than 16 ($R'>1$) and $\delta$ assigning more weight to the power for the secondary the wBH is superior.

Let us now consider the case that any of the secondary endpoints being tested now can replace in terms of its importance the primary one in later phases. Therefore, making a type I error for a secondary endpoint is almost as crucial as for the primary. This should be reflected with R close to 1, say in the range 1–2. The stronger we assess that the primary endpoint reflects the superiority of the treatment better than any of the secondary ones, the higher R should be. Power consideration should be similar for the primary and any of the secondary ones so we choose $\delta\sim 1$, in order to calculate $\Pi_{\delta}$. Here the use of HWF is recommended if we assess that a few of the secondary endpoints might be affected and most of them not substantially so. Otherwise use wBH.

If we consider a case quite similar to the above in the sense that any of the secondary endpoints can replace the primary, yet there is still an advantage in rejecting the primary endpoint rather than a secondary possibly because of economic, or regulatory considerations. That should be reflected with the same choice of R as above, but more emphasis should be given in the power evaluation to the rejection of the primary, i.e. $\delta > 1$, possibly much larger. The conclusions will remain as before, and the region where the HWF has more power increases. The advantage stems from the higher power to discover the effect for the primary endpoint.

When the primary endpoint indeed determines the success in the sense that its failure undermines further testing, $R$ should be much larger, at least $R=S$, (so $R'=1$), but possibly much higher. The secondary endpoints may then signal important additional benefits, but they should at most amount when summed up to the weight of the primary, and quite likely they should have even smaller weight $R'=1, 2, 4$ or even 10. More emphasis should be given in the power evaluation to the rejection of the primary, of course, but the rejection of the secondary endpoints cannot be ignored, as it signals reliable additional benefits. Note that here, even the wBH may have more power, but it does not offer good enough protection for the statements about the secondary. Hence from a conservative point of view, we would argue that if a large $R$ is chosen the HWF should be used.

We emphasize that the above properties should be considered prior to conducting the trial, and the choice of the method, either wBH or HWF, and the weights ratio $R$, should be made at the design stage, as is the practice regarding every other decision regarding the statistical analysis. The details should therefore be specified in the protocol of the trial.

## 7. Discussion

Our analysis of the sample of clinical trials reported by the *New England Journal of Medicine* shows that multiplicity issues are hardly addressed, except by the practice of designating primary endpoints, and in 84% of them a single primary endpoint was designated. In another 10%, two primary endpoints were designated and we do have a result for this case. Of a similar nature is our decision to address only the case of equal weights to the secondary endpoints. An expression for the maximum error-rate for this case under HWF procedure is available, but assigning these unequal weights will need more reflection on their clinical meaning. Addressing these two issues in the current work would have made it longer and much more complex. We therefore defer these two issues for later publication.

Kaplan (2008), written in collaboration with American Food and Drug administration reveals an increase in Phase III failure rates from 30% to 50% in recent years. One of the reasons offered for this finding is the lack of correction for the multiplicity issue. In 2009, the FDA issued a directive concerning necessary multiplicity correction required for significance levels of secondary endpoints in studies concerning surgical ablation devices for treatment of atrial fibrillation, stating that “If you intend to present comparisons between groups for a secondary effectiveness endpoint in your labeling, your protocol should include a pre-specified hypothesis and an adjustment for multiplicity, as appropriate.” This directive further indicates that control of the some error criterion for the secondary endpoints on top of the concern over the primary ones is getting attention by the regulatory agencies. Our opinion is that weighted false discovery control is an appropriate answer to this concern.

Genovese *and others* (2006) have offered another weighted FDR procedure, where hypotheses that get larger weights have increased probability of being rejected, at the (small) expense of reducing the power for the low weighted hypotheses. Such weights may reflect prior probabilities about the alternative being correct, and therefore can play a role in Bayesian analysis. Their procedure controls the regular FDR rather than a weighted one; this is sometimes an advantage but in the current setting it does not reflect the difference in importance assigned to primary versus secondary endpoints. We have also limited our detailed simulations work to small to medium size number of secondary endpoints (below 100). The reason is that this range reflects the number of endpoints studied in a typical clinical trial. This is unlike other areas where FDR is being used for guiding multiple testing, where the number of tests is at least in the thousands. Therefore, we have not put up for comparisons empirical Bayes procedures that are appropriate for the larger problems.

In a nutshell, the weighted procedures studied here are especially important when the primary endpoint tested at Phase II of clinical trial has low to moderate power of discovery, possibly comparable to some of the secondary ones. This is a common situation, and the procedures offer risk diversification in that it more equally disperses the power of discovery assigned to primary and secondary endpoints as compared to other procedures. Yet they offer protection from the drawback of selective inference, both to the patients and to the investigator, and especially the HWF does so.

Attentive employment of the weighted procedures by assigning “correct” weights to the various hypothesis or thoughtful application of the ratio $R$ could increase the established extent of power. Therefore, an important research goal would be the development of a methodology aimed at proper and relevant weighting in medical research.

The HWF procedure suggested herein may be easily extended and applied to different types of research that set hypotheses that may be divided into different importance levels. Indeed, many fields of research other than clinical trials set a single primary hypothesis above other tested hypotheses, allowing for the application of the HWF procedures in a broad variety of research and application fields.

In some applications, the weights may reflect a monetary value that can be assigned to the discoveries. In other research fields, such as genomics, the growing number of comparisons may be subdivided into two or more levels. The employment of a generalization of the HWF procedure to more than two levels may offer a benefit, by increasing the general power as well as the power of discovery of lower level hypotheses.

## Supplementary material

Supplementary material is available at http://biostatistics.oxfordjournals.org.

## References

[B1] Benjamini Y. and Hechtlinger Y. (2014). Discussion: an estimate of the science-wise false discovery rate and application to the top medical literature. Biostatistics 15, 13–16. 24068247 10.1093/biostatistics/kxt032

[B2] Benjamini Y. and Heller R. (2008). Screening for partial conjunction hypotheses. Biometrics 64, 1215–1222. 18261164 10.1111/j.1541-0420.2007.00984.x

[B3] Benjamini Y. and Hochberg Y. (1995). Controlling the false discovery rate: a practical and powerful approach to multiple testing. Journal of the Royal Statistical Society. Series B, Statistical Methodology 57, 289–300.

[B4] Benjamini Y. and Hochberg Y. (1997). Multiple hypotheses testing with weights. Scandinavian Journal of Statistics 24, 407–419.

[B5] Benjamini Y. and Kling Y. (1999). A look at statistical process control through the p-values. Technical Report, Tel Aviv, Israel: Tel Aviv University.

[B6] Benjamini Y. and Yekutieli D. (2001). The control of the false discovery rate under dependency. Annals of Statistics 29, 1165–1188.

[B7] Dmitrienko A., Offen W. W. and Westfall P. H. (2003). Gatekeeping strategies for clinical trials that do not require all primary effects to be significant. Statistics in Medicine 22, 2387–2400. 12872297 10.1002/sim.1526

[B8] Dmitrienko A. O., Tamhane, A. C. and Wiens, B. L. (2008). General multistage gatekeeping procedures. Biometrical Journal 50, 667–677. 18932130 10.1002/bimj.200710464

[B9] Genovese C. R., Roeder K. and Wasserman L. (2006). False discovery control with p-value weighting. Biometrika 93, 509–524.

[B10] Ghosh S., Goldin E., Gordon F. H., Malchow H. A., Rask-Madsen J., Rutgeerts P, Vyhnálek P., Zádorová Z., Palmer T. and Donoghue S. (2003). Natalizumab for active Crohn’s disease. New England Journal of Medicine 348, 24–32. 12510039 10.1056/NEJMoa020732

[B11] Guilbaud O. (2007). Bonferroni parallel gatekeeping—transparent generalizations adjusted p-values and short direct proofs. Biometrical Journal 49, 917–927. 17726713 10.1002/bimj.200610361

[B12] Ioannidis J. P. A. (2005). Why most published research findings are false. PLoS Medicine 2, 124. 10.1371/journal.pmed.0020124 PMC1182327 16060722

[B13] Jager L. and Leek J. (2014). An estimate of the science-wise false discovery rate and application to the top medical literature. Biostatistics 15, 1–12. 24068246 10.1093/biostatistics/kxt007

[B14] Kaplan M. M. (2008). Current dilemma in drug development—increasing failure rate of investigational drugs in phase3 clinical.

[B15] Shaffer J. P. (1995). Multiple hypothesis testing: a review. Annual Review of Psychology 46, 561–584.

[B16] Simes R. J. (1986). An improved procedure for multiple tests of significance. Biometrika 73, 751–754.

[B17] Yekutieli D., Reiner-Benaim A., Elmer G. L., Kafkafi N., Letwin N. E. Lee, N. H. and Benjamini Y. (2006). Approaches to multiplicity issues in complex research in microarray analysis. Statistica Neerlandica 60, 414–437.

